# Supplementary material for: Multi-scale computational study of the mechanical regulation of cell mitotic rounding in epithelia
Source: PLoS Comput Biol. 2017 May 22;13(5):e1005533. doi: 10.1371/journal.pcbi.1005533 (PMC5460904; doi:10.1371/journal.pcbi.1005533)
Supplement: S1 Appendix — (PDF) [file pcbi.1005533.s001.pdf]

S1 Appendix: Computational implementation and computational cost of the Epi-Scale model

S1.1 Parallelized GPU implementation of the Epi-Scale model

Even simple tissues consist of thousands of cells. For example, *Drosophila* prepupal wing disc pouch grows to about 5,000 cells and the whole wing disc includes 30,000-50,000 cells [1,2]. A multi-scale model with submodels for separately simulating membrane, cytoplasm, and cell-cell adhesion would require implementation on clusters of graphical processing units (GPUs) to reduce the very high computational cost of simulations.

A flowchart outlining the computational implementation of the Epi-Scale model is shown in Figure S1.1. This developed cell-based subcellular element model (SEM) assumes two types of interaction forces that act on the specific element: 1. Intracellular forces act on coarse-grained elements within a single cell; and 2. Intercellular forces act between membrane-bound elements of neighboring, interacting cells. The intracellular forces represent “bounded” interactions that are computed without a requirement to search for interacting elements. The spring force between two neighboring membrane elements of the same cell is an example of such a “bounded” interaction.

Simulating intercellular “unbounded” interactions is more computationally expensive since it depends on the relative distances between two elements. For example, the distances between two membrane elements must be calculated first to determine whether or not a pairwise adhesion interaction exists between the two membrane nodes.

The number of computational operations for implementing bounded interactions increases linearly with the increase of the number of cells and, hence, bounded interactions have relatively low computational cost. Without implementing algorithm optimization, unbounded interactions are computationally more expensive than bounded interactions because of the cost associated with finding closest neighbors to a given cell. For example, the number of computational operations (N_{Op}) for calculating the adhesive forces between membrane nodes of different cells are of the order of:

$$N_{Op} = \mathcal{O}(N_{cell} \times N_{memb})^2 \qquad \text{(S1.1)}$$

where N_{cell} is the number of cells and N_{memb} is the number of membrane nodes per cell. S1.1 approximates the total number of possible operations that are needed to simulate all membrane-membrane interactions at every simulation time step. Therefore, the total computational time is of the order of:

$$T_{total} \propto (N_{cell} N_{memb})^2 \qquad \text{(S1.2)}$$

where T_{total} is the total computational time for simulating N_{cell} number of cells with subcellular resolution. To decrease the computational time, we have used CUDA programming platform to run the simulations on the GPUs [3] (described in S1.2). Also, we have used an efficient search algorithm to reduce the number of required operations (described in S1.3).

S1.2 Reduction of computational time with GPU implementation

Mathematical operations are performed in parallel on GPUs, which consist of thousands of threads. In the CUDA programming language which is used for GPUs, operations are written in vector form, which permits calculations to be distributed among the threads. This results in a dramatic reduction of the computational time (compared with Expression S1.2):

$$T_{total} \propto \frac{B_{comm}(N_{cell}N_{memb})^2}{N_{Th}} \quad \text{(S1.3)}$$

where N_{Th} is number of threads in GPUs, and B_{comm} is the computational time between the threads in a GPU cluster. Thus, the total simulation time decreases with an increase in the number of threads in the GPU cluster.

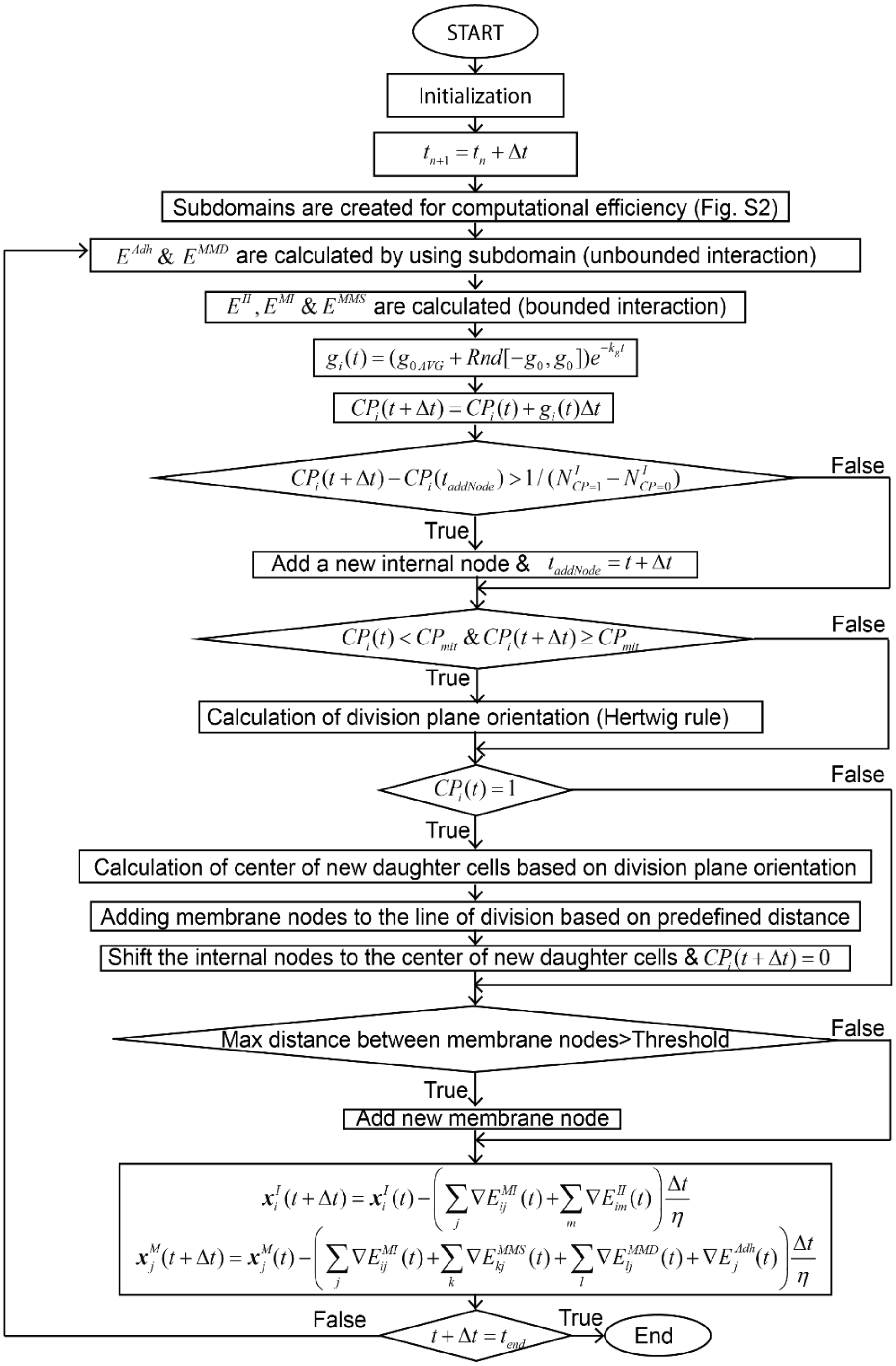

Fig S1.1 Flowchart of the Epi-Scale model implemented on the GPU cluster. Calculation of $\boldsymbol{E^{Adh}}$ and $\boldsymbol{E^{MMD}}$ on the GPU cluster is optimized by using an efficient search algorithm as described in Section S1.3.

S1.3 Search algorithm to reduce computational cost of computing unbounded interactions

The number of computational operations is proportional to the square of the number of simulated cells (Expression S1.3). We developed a search algorithm to further decrease the computational time that provides an asymptotically linear number of operations with respect to the number of simulated cells and nodes. The total area occupied by cells is divided into M square subdomains. The size of the subdomains is determined based on the longest distance at which two elements can interact with each other. Fig. S1.2 shows a representative simulation with 17 x 17 subdomains. Since cell-cell adhesion interactions are short range, the search algorithm for implementing corresponding interaction forces is limited to only neighbouring sub-domains. The number of neighbouring subdomains is independent of the number of cells in the tissue. There are eight neighbouring subdomains for each internal subdomain (Fig. S1.2). Therefore, the algorithm reduces the total number of operations by searching only within this 8 neighboring subdomains. For example, search for the elements which are inside the blue subdomain in Fig. S1.2 is only performed within the eight neighboring subdomains, numbered from 1 to 8, for applying unbounded forces.

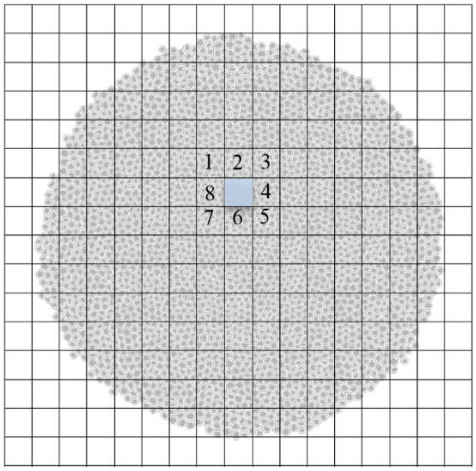

Fig S1.2 Subdivision of the simulation domain. Domain is occupied by the growing tissue consisting of 1200 cells, into smaller square sub-domains optimizes the search algorithm for applying forces between cells. Only cells within the eight indicated neighboring sub-domains are used for applying unbounded interaction forces for the cells located in the blue subdomain.

The parallel array expansion and parallel pre-fixed sum, provided by the Thrust library, are used in the GPU implementation of the algorithm as shown in Fig. S1.3.

```
for element_i in SceElements:
    i_subdomain_id = find_subdomain_id(element_i);
    subdomain_id_arr[i] = i_subdomain_id;
    element_id_arr = i;
sort_by_key(subdomain_id_arr, element_id_arr);
(tab_expa, ele_expa)=expand_trans(subdomain_id_arr, element_id_arr);
(upperBounds, lowerBounds) = find_bounds(tab_expa)
for element_i in element_id_arr:
    for j from lowerBounds[i] to upperBounds[j]:
        compute_interaction(element_i, SceElements[ele_expa[j]])
```

Fig S1.3 GPU neighbor sub-domain search algorithm. First, neighboring sub-domains are identified for each element. Then, within these subdomains the magnitudes of unbounded interacting forces are calculated.

The time complexity of the algorithm is reduced to $\boldsymbol{O(N_{cell}N_{memb})}$ by implementing simulation domain sub-division. Therefore, using the search algorithm reduces the total computational time to:

$$T_{total} \; \alpha \; \frac{N_{NS}B_{comm} \; O(N_{cell}N_{memb})}{N_{Th}} \tag{S1.4}$$

where $\boldsymbol{N_{NS}}$ is the number of neighboring subdomains. Comparison of the expressions S1.1 and S1.4 shows that GPU implementation with the search algorithm reduces simulation time from being proportional to the square of the number of simulated cells to being proportional to the number of simulated cells.

S1.4 Comparison of computational costs of the Epi-Scale model and other models

The Epi-Scale model gives detailed information about mechanical properties of a single cell, but it comes with relatively higher computational cost compared to VBM and CPM models. The numbers of cells vs. the computational cost in simulation hours are plotted in Fig. S1.4. The computational cost refers to time takes to numerically simulate the growth of the epithelial tissue until a given number of cells. The model starts with seven cells at time zero and cells grow and divide to reach over one thousand of cells (Fig. 3 in the main text). Fig. S1.4 shows a linear relationship between the computational cost and number of cells. This relationship is in agreement with Expression S1.4 where the simulation time ($\boldsymbol{T_{total}}$) is proportion to the number of cells ($\boldsymbol{N_{cell}}$).

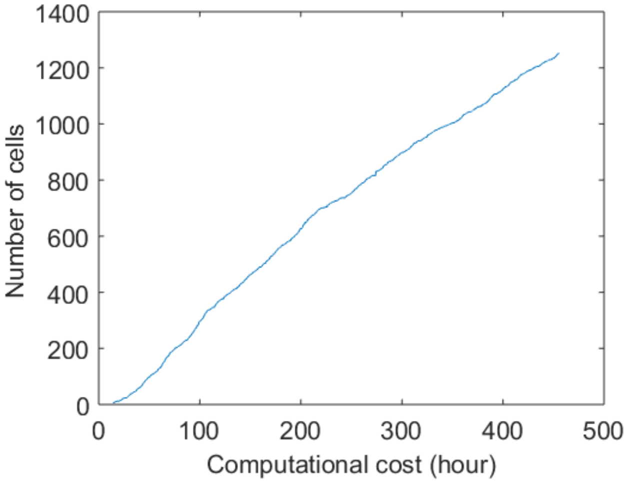

Fig S1.4 Computational cost analysis of the Epi-Scale model.

The computational cost of simulating tissue growth in the Epi-Scale model is approximately compared to the computation cost of simulating tissues with a Cellular-Potts model (CPM) described in the literature (Table S1.1) [4] as follows. In the CPM model, 10000 cells are simulated which are growing in cellular size but not in number. In the Epi-Scale model, the simulated tissue grows from 7 to 1200 cells.

Table S1.1 Comparison of computational costs of the Epi-Scale and CPM.

**Cell modelling approach**	**SEM (Epi-Scale)**	**CPM [4]**
Method of simulation (Cell growth test)	1 GPU (GeForce GTX 780 Ti)	25 CPU (AMD 64 bit 2.2 GHz)
Size of marching time step	0.003 seconds	1 Monte-Carlo iteration
Maximum Number of cells	1200	10000
"Simulation time" for one step for maximum number of cells	0.036 seconds	0.059 for 1 Monte-Carlo step
"Simulation time" normalized by number of cells	3.0×10^{-5} seconds	5.9×10^{-6} seconds

Table S1.1 shows that computational cost of the Epi-Scale model is five times higher than computational cost of the CPM after it is normalized by the number of cells. One of the key differences between these two approaches is that in the Epi-Scale model each sub-cellular element is free to move in any direction, while in the CPM model nodes belong to a fixed lattice and change their state. Therefore, relatively higher computational cost of the Epi-Scale model enables a more mechanically realistic description of cell shapes (see S1 Movie). This increased mechanical resolution also makes Epi-Scale more biologically relevant in comparison with the vertex based models, which assume cell shapes as polygons.

References

1. Garcia-Bellido A, Merriam JR. Parameters of the wing imaginal disc development ofDrosophila melanogaster. Dev Biol. 1971;24: 61–87. doi:10.1016/0012-1606(71)90047-9

2. Martin FA, Herrera SC, Morata G. Cell competition, growth and size control in the Drosophila wing imaginal disc. Development. 2009;136: 3747–3756. doi:10.1242/dev.038406

3. Dematté L, Prandi D. GPU computing for systems biology. Brief Bioinform. 2010;11: 323–333. doi:10.1093/bib/bbq006

4. Chen N, Glazier JA, Izaguirre JA, Alber MS. A parallel implementation of the Cellular Potts Model for simulation of cell-based morphogenesis. Comput Phys Commun. 2007;176: 670–681. doi:10.1016/j.cpc.2007.03.007
