# Supplementary material for: Multi-scale computational study of the mechanical regulation of cell mitotic rounding in epithelia
Source: PLoS Comput Biol. 2017 May 22;13(5):e1005533. doi: 10.1371/journal.pcbi.1005533 (PMC5460904; doi:10.1371/journal.pcbi.1005533)
Supplement: S2 Appendix — (PDF) [file pcbi.1005533.s002.pdf]

S2 Appendix: Potential energy functions used in the Epi-Scale model

The following potential energy functions are used in the Epi-Scale model.

$$E_{ij}^{MI} = \left[U^{MI} \exp\left(-\frac{|x_i - x_j|}{\xi^{MI}}\right) - W^{MI} \exp\left(-\frac{|x_i - x_j|}{\gamma^{MI}}\right)\right] \quad \text{(S2.1)}$$

$$E_{ij}^{II} = \left[U^{II} \exp\left(-\frac{|x_i - x_j|}{\xi^{II}}\right) - W^{II} \exp\left(-\frac{|x_i - x_j|}{\gamma^{II}}\right)\right] \quad \text{(S2.2)}$$

$$E_{ij}^{MMD} = \left[U^{MMD} \exp\left(-\frac{|x_i - x_j|}{\xi^{MMD}}\right) - W^{MMD} \exp\left(-\frac{|x_i - x_j|}{\gamma^{MMD}}\right)\right] \quad \text{(S2.3)}$$

$$E_{ij}^{MMS} = \sum_{i=j-1}^{i=j+1} \frac{1}{2} k^{Stiff} (|x_i - x_j| - L^{Stiff})^2 + \alpha_{ij} \frac{1}{2} k^{Tor} \theta_{ij}^2 \quad \text{(S2.4)}$$

$$E_i^{Adh} = \begin{cases} \max(\frac{1}{2} k^{Adh} (|x_i - x_{i-pair}| - L_{min}^{Adh})^2, 0) & |x_i - x_{i-pair}| \leq L_{max}^{Adh} \\ 0 & |x_i - x_{i-pair}| > L_{max}^{Adh} \end{cases} \quad \text{(S2.5)}$$

The coefficients of the Morse potential function are given in Table 2 of the main text. α_{ij} is calculated based on the position of membrane nodes [1]. x_{i-pair} is the position of the closest membrane node of neighbouring cell to the membrane node i.

References

1. Hui K, Lin G, Pan W. Understanding the mechanisms of sickle cell disease by simulations with a discrete particle model. Comput Sci Discov. 2013;6: 015004. doi:10.1088/1749-4699/6/1/015004
