# Supplementary material for: Multi-scale computational study of the mechanical regulation of cell mitotic rounding in epithelia
Source: PLoS Comput Biol. 2017 May 22;13(5):e1005533. doi: 10.1371/journal.pcbi.1005533 (PMC5460904; doi:10.1371/journal.pcbi.1005533)
Supplement: S3 Appendix — (PDF) [file pcbi.1005533.s003.pdf]

S3 Appendix: Metrics for determining polygon class, roundness of cell shape and internal pressure of a cell

S3.1 Polygon class

The polygon class for a cell is determined by counting the number of its neighbors. Two cells are considered as neighbor if the minimum distance between their membrane nodes is less than half-length of an average cell diameter. Cells at the boundary of the simulated tissue domain are excluded from the polygon class calculation or statistical analysis.

S3.2 Cell area

Cell area is calculated by summing up areas of the triangles with vertices at the membrane nodes and center of the cells as shown in Figure S3.1.

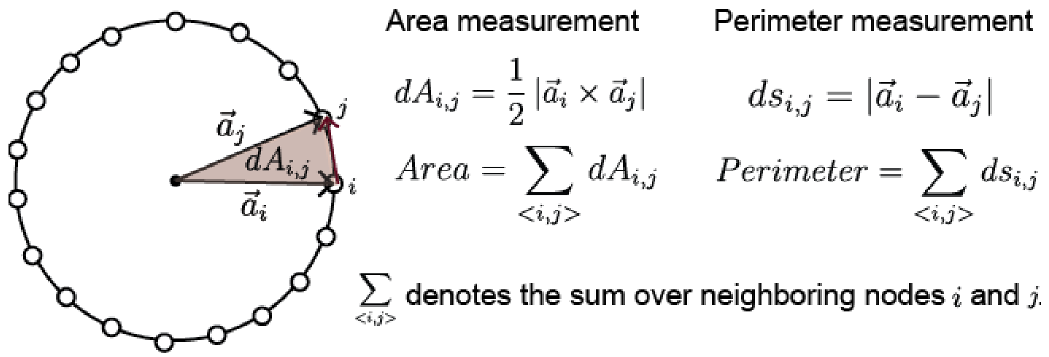

Fig S3.1. Diagram demonstrating measurement of the cell area and perimeter.

S3.3 Cell roundness

The isoperimetric quotient, or roundness (R) [1], was used to quantify shapes of individual cells. The expression for the isoperimetric quotient in general is as follows:

$$R = 4\pi A/Pe^2 \quad \text{(S3.1)}$$

where A and Pe are the area and perimeter of the cell, respectively. The measurements of the cell area and perimeter are depicted in Fig S3.1. Interphase cells have a polygon shape. Cell roundness increases as they progress through the mitotic phase. The normalized roundness, R_{norm}

$$R_{norm} = (R - R_p)/(1 - R_p) \quad \text{(S3.2)}$$

is equal to 0 for a perfect hexagon and $R_{norm} = 1$ to a perfect circle. R_p is the roundness for a polygon with six sides:

$$R_p = \frac{\pi}{ntan\left(\frac{\pi}{6}\right)} \quad \text{(S3.3)}$$

S3.4 Calculation of the increase of cytoplasmic pressure in mitotic cells

The increase in internal pressure of the cell in mitotic phase is calculated in a way similar to the experimental setup frequently used for measuring pressure of mitotic cells [2,3]. The initial condition of simulation is shown in Fig S3.2a where a cell in interphase stage is placed between a plate and cantilever in resting condition without applying any force to the cell.

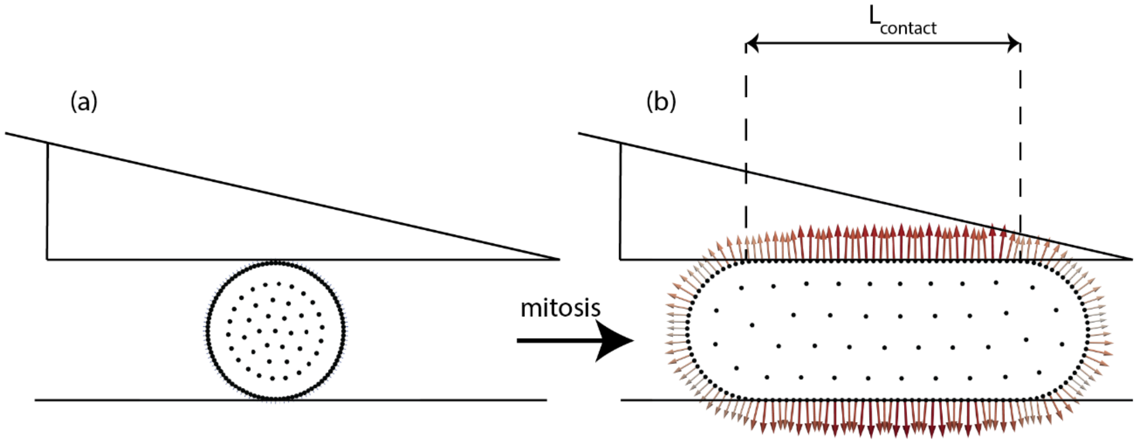

Fig S3.2. Estimation of the increase in the internal pressure of a cell. (a) Cell in interphase stage, (b) cell in the mitotic phase stage.

As the cell starts to undergo mitotic phase, the internal pressure inside the cell is increased to expand the cell and push against cantilever. The cantilever is fixed resulting in a pressure increase in the cell. To calculate increase in pressure, the forces applied from the internal nodes to membrane nodes of the cells that are in contact with cantilever are summed up and then divided by the length of the contact line shown in Fig S3.2b.

$$\Delta P = \frac{\sum_{i=1}^{m_c} F_i^N}{L_{contact}}, \qquad \text{(S3.4)}$$

Here ΔP is the increase in the internal pressure of the cell in mitotic phase, m_c is the number of membrane nodes of the cell in contact with cantilever, and F_i^N are the magnitudes of forces applied from internal nodes to the membrane node i in normal direction (Fig S3.2b).

References

1. Croft HT, Falconer K, Guy RK. Unsolved Problems in Geometry: Unsolved Problems in Intuitive Mathematics. Corrected edition. New York: Springer; 1994.

2. Stewart MP, Helenius J, Toyoda Y, Ramanathan SP, Muller DJ, Hyman AA. Hydrostatic pressure and the actomyosin cortex drive mitotic cell rounding. Nature. 2011;469: 226–230. doi:10.1038/nature09642

3. Cattin CJ, Düggelin M, Martinez-Martin D, Gerber C, Müller DJ, Stewart MP. Mechanical control of mitotic progression in single animal cells. Proc Natl Acad Sci. 2015;112: 11258–11263. doi:10.1073/pnas.1502029112
