# Supplementary material for: Multi-scale computational study of the mechanical regulation of cell mitotic rounding in epithelia
Source: PLoS Comput Biol. 2017 May 22;13(5):e1005533. doi: 10.1371/journal.pcbi.1005533 (PMC5460904; doi:10.1371/journal.pcbi.1005533)
Supplement: S4 Appendix — (PDF) [file pcbi.1005533.s004.pdf]

S4 Appendix: Detailed analysis of the Aboav-Weaire law

The Aboav-Weaire law states that the average polygon class of a cell's neighbors decreases as the cell's polygon class increases. Fig. S4.1 shows that our simulation results satisfy the Aboav-Weaire law. The fraction of cells for each polygon class is also shown in the Table S4.1.

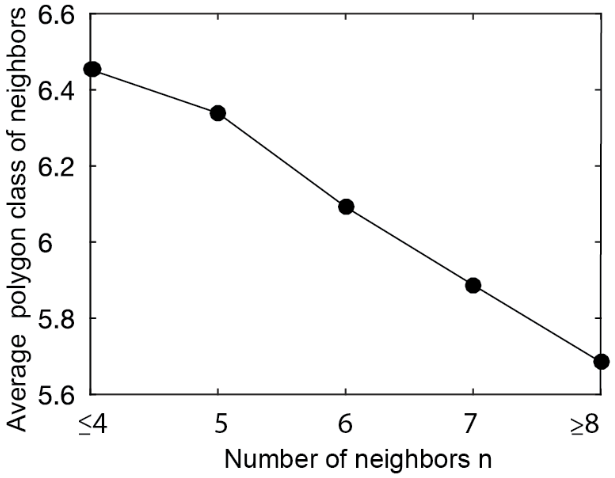

Fig. S4.1. The average polygon class of neighbouring cells decreases as the cells gain more sides. This is consistent with the Aboav-Weaire law.

Table S4.1. Polygon class distribution of neighboring cells.

	**Fraction of neighbors with given polygon class**				
**Polygon class of cell**	**≤4**	**5**	**6**	**7**	**≥8**
**≤4**	0.032	0.138	0.324	0.364	0.142
**5**	0.036	0.162	0.417	0.279	0.100
**6**	0.028	0.234	0.456	0.211	0.069
**7**	0.036	0.320	0.432	0.178	0.033
**≥8**	0.054	0.373	0.453	0.108	0.106
