# Supplementary material for: Multi-scale computational study of the mechanical regulation of cell mitotic rounding in epithelia
Source: PLoS Comput Biol. 2017 May 22;13(5):e1005533. doi: 10.1371/journal.pcbi.1005533 (PMC5460904; doi:10.1371/journal.pcbi.1005533)
Supplement: S5 Appendix — (PDF) [file pcbi.1005533.s005.pdf]

S5 Appendix: Calibration of the adhesion between neighboring cells

Fig S5.1 shows the experimental setup used to obtain data to calibrate submodel describing adhesivity between neighboring cells in the Epi-Scale. The data reported for transfected S180 cells are used. Cadherin transmembrane proteins are shown to be responsible for adhesivity of these cells, which is similar to the adhesivity of the epithelial cells of *Drosophila* wing disc. Fig S5.1a shows two adhered cells in vitro [1]. Cells are pulled away by using a pipette and the force required to detach the cells is measured (Fig S5.1b). Simulation of this experiment is used to calibrate value of the parameter that controls the strength of cell-cell adhesion in the computational model. In Fig S5.1c, two adhered cells are shown at rest. The magnitude of the force required to detach the cells is estimated in our simulations and compared with the experimental measurements. The reported forces for the detachments of the cells in the experiment are of the order of 10^2 nN [1]. The measured force is normalized by dividing by the length of the contact line (Fig S5.1a). Order of the detachment force per unit length of the contact line between two adhering cells in experiments is as follows:

$$\boldsymbol{O}\left(\frac{\boldsymbol{F}_{\boldsymbol{in\ vitro}}}{\boldsymbol{L}_{\boldsymbol{in\ vitro}}}\right) = \frac{\boldsymbol{O}(\boldsymbol{F}_{\boldsymbol{vitro}})}{\boldsymbol{O}(\boldsymbol{L}_{\boldsymbol{vitro}})} = \frac{\boldsymbol{O}(\mathbf{10^2}\ \boldsymbol{nN})}{\boldsymbol{O}(\mathbf{10}\ \boldsymbol{\mu m}\ )} = \boldsymbol{O}(\mathbf{10}\ \boldsymbol{nN}/\boldsymbol{\mu m}) \quad \text{(S5.1)}$$

The range of the adhesivity parameter in Epi-Scale model was calibrated so that the order of detachment force per unit length of the contact line between two adhering cells in simulations would be the same as in experiments:

$$\frac{\boldsymbol{O}(\boldsymbol{F}_{\boldsymbol{in\ silico}})}{\boldsymbol{O}(\boldsymbol{L}_{\boldsymbol{in\ silico}}\ )} = \boldsymbol{O}(\mathbf{10}\ \boldsymbol{nN}/\boldsymbol{\mu m}) \quad \text{(S5.2)}$$

The adhesivity between the cells is modeled as a cumulative effect of individual springs connecting pairs of nodes in the membranes of neighboring cells. The stiffness of the springs is calibrated so that the force required to detach two cells in simulation is of the order of 10 nN. Alternatively, normalizing the data provided in [2] for MDCK cells by the contact length, results in a detachment force with a similar order of magnitude.

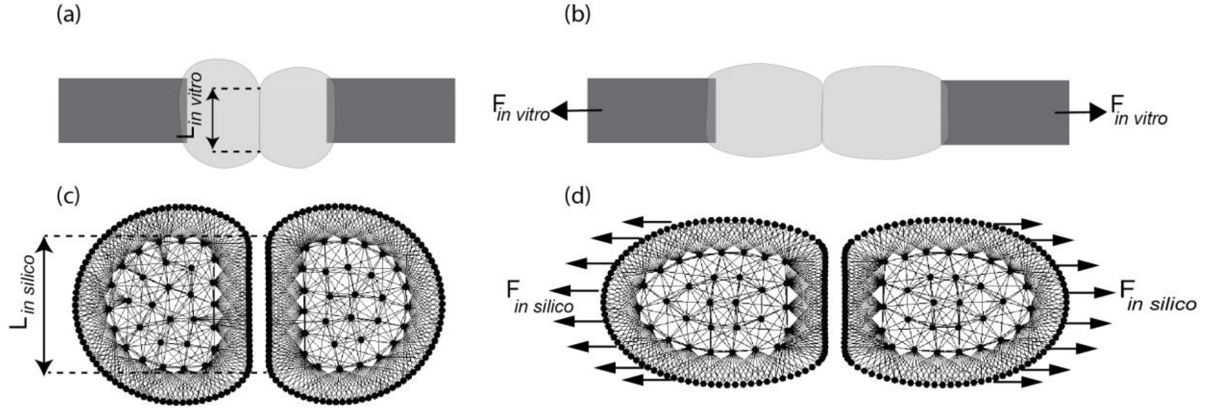

Fig S5.1. Stretching test for calibrating adhesivity force parameters. Adhesivity test in vitro for the transfected S180 cells, (a) before applying stretching force (b) after applying stretching

forces [1]. Adhesivity test in Epi-Scale model with (c) no stretching force is applied or (d) with the application of an external stretching force.

References

1. Chu Y-S, Thomas WA, Eder O, Pincet F, Perez E, Thiery JP, et al. Force measurements in E-cadherin–mediated cell doublets reveal rapid adhesion strengthened by actin cytoskeleton remodeling through Rac and Cdc42. J Cell Biol. 2004;167: 1183–1194. doi:10.1083/jcb.200403043

2. Sim JY, Moeller J, Hart KC, Ramallo D, Vogel V, Dunn AR, et al. Spatial distribution of cell–cell and cell–ECM adhesions regulates force balance while main-taining E-cadherin molecular tension in cell pairs. Mol Biol Cell. 2015;26: 2456–2465. doi:10.1091/mbc.E14-12-1618
