# Supplementary material for: Multi-scale computational study of the mechanical regulation of cell mitotic rounding in epithelia
Source: PLoS Comput Biol. 2017 May 22;13(5):e1005533. doi: 10.1371/journal.pcbi.1005533 (PMC5460904; doi:10.1371/journal.pcbi.1005533)
Supplement: S6 Appendix — (PDF) [file pcbi.1005533.s006.pdf]

S6 Appendix: Transgenic fly lines, microscopy and image processing

S6.1 Fly lines and microscopy

Drosophila line maintenance, wing disc dissection, culture and imaging were performed as previously described [1,2]. The DE-cadherin::GFP line [3] as well as a GFP-tagged line expression Myosin II, Sqh::GFP (gift from A. Martin, Fig. 1) were used to image representative wing discs on a Nikon Eclipse Ti confocal microscope (Nikon Instruments) with a Yokogawa spinning disc system (Andor Technology). Multiple z-slices were z-projected discs were imaged using a 100x magnification objective (NA 1.49). For time-lapse videos of mitotic rounding, wing imaginal discs were dissected from 3rd instar larvae and cultured in an *ex vivo* imaging chamber in WM1, a media optimized for long-term culture of *Drosophila* wing discs.

S6.2 Image processing and analysis

Time-series of cell boundaries were analyzed in the open source software Epitools [4]. Epitools was used to segment and track 22 cells undergoing mitosis and to obtain masks before and during mitotic rounding. The simulation was calibrated to area and roundness values extracted from these cell-shape masks.

References

1. Zartman J, Restrepo S, Basler K. A high-throughput template for optimizing Drosophila organ culture with response-surface methods. Development. 2013;140: 667–674. doi:10.1242/dev.088872

2. Restrepo S, Zartman JJ, Basler K. Cultivation and Live Imaging of Drosophila Imaginal Discs. Methods Mol Biol Clifton NJ. 2016;1478: 203–213. doi:10.1007/978-1-4939-6371-3_11

3. Huang J, Zhou W, Dong W, Watson AM, Hong Y. Directed, efficient, and versatile modifications of the Drosophila genome by genomic engineering. Proc Natl Acad Sci. 2009;106: 8284–8289. doi:10.1073/pnas.0900641106

4. Heller D, Hoppe A, Restrepo S, Gatti L, Tournier AL, Tapon N, et al. EpiTools: An Open-Source Image Analysis Toolkit for Quantifying Epithelial Growth Dynamics. Dev Cell. 2016;36: 103–116. doi:10.1016/j.devcel.2015.12.012
