# Supplementary material for: Multi-scale computational study of the mechanical regulation of cell mitotic rounding in epithelia
Source: PLoS Comput Biol. 2017 May 22;13(5):e1005533. doi: 10.1371/journal.pcbi.1005533 (PMC5460904; doi:10.1371/journal.pcbi.1005533)
Supplement: S7 Appendix — (PDF) [file pcbi.1005533.s007.pdf]

S7 Appendix: Mitotic rounding effects on cell-cell rearrangements

Mitotic rounding is necessary for spindle formation and proper division of mitotic cell to two daughter cells. Mitotic rounding also contributes to tissue morphogenesis. For example Kondo and Hayashi showed that mitotic cell rounding can accelerate epithelial invagination [1]. Cell-cell rearrangements, or T1 transitions, is another extremely important morphogenetic process. T1 transitions occurs when two cells that were previously neighbors are no longer neighbors, and two cells that were previously not neighbors become neighbors (Fig S7.1a). T1 transitions are extremely important in many morphological process such as germ band extension [2]. Our simulation results show that rapid increase in cell area of the mitotic cells can result in T1 transitions with neighboring cells during rounding. The rapid increase in the size of the mitotic cells pushes the neighboring cells, which result in T1 transitions. Fig. S7.1b is an example of T1 transition occurring next to a mitotic cell.

Interestingly, pressure can increase or decrease mitotic roundness (R_{norm}) depending on the relative levels of adhesion and cortical stiffness. In some cases very non-round mitotic cell phenotypes (star-shaped) can be generated when cortical stiffness is decreased, which is similar to what is reported for cultured cells with ERM regulating proteins [3], required for cross-linking cortical actin, are perturbed (Fig. S7.1c).

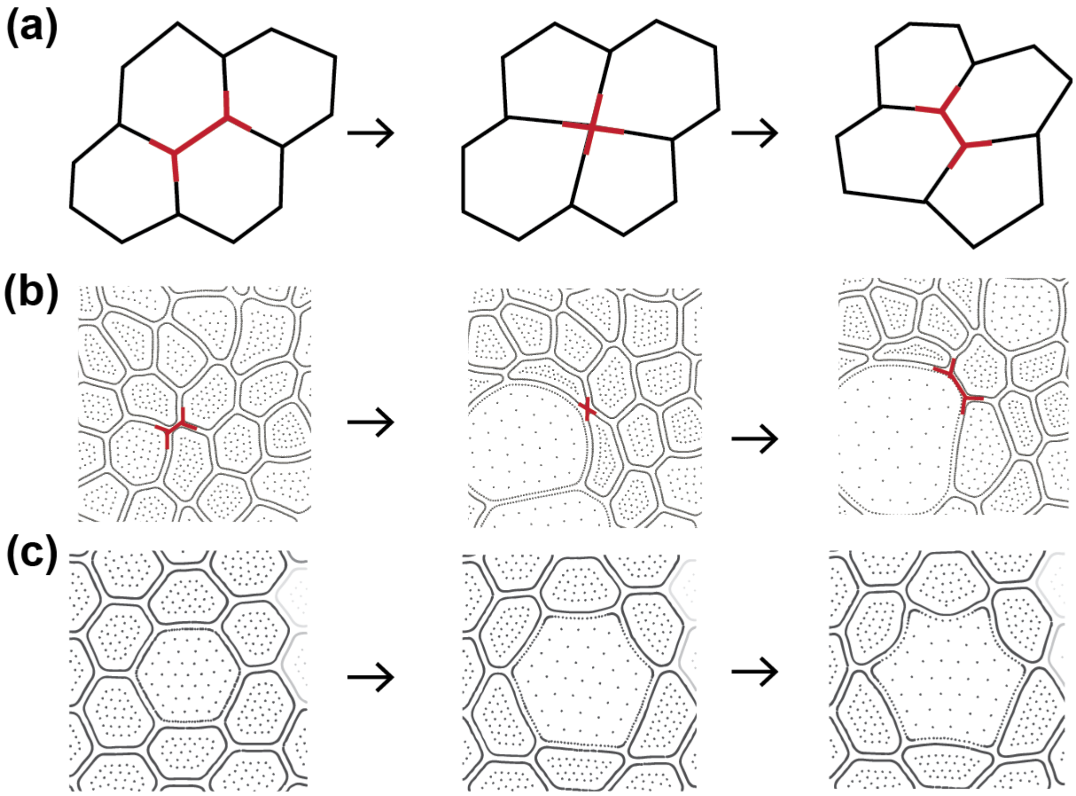

Fig S7.1 Mitotic rounding can drive T1 transitions (cell rearrangements). (a) schematic of T1 transition. (b) T1 transition occurs around a mitotic cell (parameter values given in Table S.3). (c) A reduced stiffness, high-pressure simulation that shows highly non-round mitotic cell shapes ($k_{mit}^{Adh} = 30\frac{nN}{\mu m}, k_{mit}^{Stiff} = 50\ nN/\mu m,\ \Delta P_{mit} = 21.7\ nN/\mu m$).

References

1. Kondo T, Hayashi S. Mitotic cell rounding accelerates epithelial invagination. Nature. 2013;494: 125–129. doi:10.1038/nature11792

2. Rauzi M, Verant P, Lecuit T, Lenne P-F. Nature and anisotropy of cortical forces orienting Drosophila tissue morphogenesis. Nat Cell Biol. 2008;10: 1401–1410. doi:10.1038/ncb1798

3. Kunda P, Pelling AE, Liu T, Baum B. Moesin Controls Cortical Rigidity, Cell Rounding, and Spindle Morphogenesis during Mitosis. Curr Biol. 2008;18: 91–101. doi:10.1016/j.cub.2007.12.051
