# Supplementary material for: Multi-scale computational study of the mechanical regulation of cell mitotic rounding in epithelia
Source: PLoS Comput Biol. 2017 May 22;13(5):e1005533. doi: 10.1371/journal.pcbi.1005533 (PMC5460904; doi:10.1371/journal.pcbi.1005533)
Supplement: S8 Appendix — (PDF) [file pcbi.1005533.s008.pdf]

S8 Appendix: Parameters used in Epi-Scale for S1 video and S2 video

S1 Video: Polygon class distribution study
The polygon class distribution is shown in the animation file. The color key for polygon classes is the same as in Fig 6b, and parameter values different than Table 2 are shown in Table S8.1.

Table S8.1. Parameters used for S1 Video. Other parameters are the same as Table 2 in the main text.

Parameter	Mitotic phase	Values during mitosis
E^{II}	U_{mit}^{II}	$19\ nN.\mu m$
	W_{mit}^{II}	$5.86\ nN.\mu m$
	ξ_{mit}^{II}	$0.54\ \mu m$
	γ_{mit}^{II}	$1.25\ \mu m$
E^{MI}	U_{mit}^{MI}	$3.81\ nN.\mu m$
	ξ_{mit}^{MI}	$0.25\ \mu m$
E^{MMS}	k_{mit}^{Stiff}	$240\ nN/\mu m$

S2 Video: Mitotic cells at high pressure lead to T1 transitions around the mitotic cells
The parameter values different than Table 2 are shown in Table S8.2. Other parameters are the same as Table 2 in the main text.

Table S8.2. Parameters used for S2 Video. Other parameters are the same as Table 2 in the main text.

Parameter	Mitotic phase	Values during mitosis
E^{II}	U_{mit}^{II}	$29.7\ nN.\mu m$
	W_{mit}^{II}	$9.16\ nN.\mu m$
	ξ_{mit}^{II}	$0.68\ \mu m$
	γ_{mit}^{II}	$1.563\ \mu m$
E^{MI}	U_{mit}^{MI}	$5.95\ nN.\mu m$
	ξ_{mit}^{MI}	$0.31\ \mu m$
E^{MMS}	k_{mit}^{Stiff}	$240\ nN/\mu m$
