# Supplementary material for: Multi-scale computational study of the mechanical regulation of cell mitotic rounding in epithelia
Source: PLoS Comput Biol. 2017 May 22;13(5):e1005533. doi: 10.1371/journal.pcbi.1005533 (PMC5460904; doi:10.1371/journal.pcbi.1005533)
Supplement: S9 Appendix — (PDF) [file pcbi.1005533.s009.pdf]

S9 Appendix: Response surface methodology

S9.1 Iteration 1: Exploration of effect of physical properties on MR

Response surface methods enable the efficient exploration of parameter space for computationally intensive models [1,2]. In a series of two iterations, we developed response surface models of mitotic area ratio ($A_{ratio} = A_{mit}/A_{inter}$) and normalized roundness (R_{norm}) roundness as a function of mitotic level set-points for cell adhesivity $\left(k_{mit}^{Adh}\right)$, cortical stiffness $\left(k_{mit}^{Stiff}\right)$, and the increase in internal pressure during mitosis (ΔP). Parameters first were varied in a three-level full factorial design (3^3 FFD) experiment encompassing a large initial range (Table S9.1). A 3^3 FFD is an experimental design where three factors are set to three different levels, and all possible combinations are tested. The range for k_{mit}^{Adh} was selected such that the change in cell-cell adhesivity during mitotic rounding ranged from no change ($k_{mit}^{Adh} = 30$) to complete loss of adhesion: ($k_{mit}^{Adh} = 0$). The range for k_{mit}^{Stiff} was selected such that cortical stiffness ranged from the minimum required for model stability ($k_{mit}^{Stiff} = 50$) to three times the interphase value ($k_{mit}^{Stiff} = 600$). The increase in pressure was varied from a pressure increase that led to almost no change in mitotic cell area ($\Delta P_{mit} = 6.1, A_{ratio} \approx 1.1$) to a pressure increase that led to a large change in mitotic cell area ($\Delta P_{mit} = 21.7, A_{ratio} \approx 5$). A quadratic regression model was fit for each of the two outputs (R and A_{ratio}), using the MATLAB function *fitlm* (Table S9.2).

S9.2 Calibration of mitotic physical parameters

R and A_{ratio} were extracted from timelapse confocal videos of E-cadherin::GFP-expressing wing discs cultured [3,4]. Epitools was used for segmentation and tracking [5], and the roundness of cells prior to division, as well as the ratio of the final area to initial area of the cell were both reported for experimental and computational data. The error between the experimental and computational R and A_{ratio} values was computed using the following function:

$$R_{error} = \frac{\left|R_{expt} - R_{sim}\right|}{R_{expt}} \quad \text{(S9.1)}$$

where R_{error} is the error in R, R_{expt} is the experimental value for R, and R_{sim} is the normalized roundness obtained in a particular simulation run.

S9.3 Iteration 2: high-resolution model of physical properties on MR

A central composite design (CCD) experiment was performed over the region where the quadratic model predicted the lowest R_{error} and $A_{ratio,error}$ to generate a high-resolution response surface model of the impacts of adhesion, stiffness, and cytoplasmic pressure on MR, (Fig 7a, Table S9.4-S9.7). A CCD is an optimal experimental design for obtaining a quadratic model of a region of parameter space with fewer simulations than an FFD.

This experiment yielded error values far lower than the initial experiment, and resulted in calibration of the model. The response surface model was augmented with additional runs that were selected with Design-Expert 7b1.1 software (Stat-Ease) using the simplex multi-objective optimization method to obtain parameter values that led to even lower error. The central point of the CCD was selected from the Pareto front as the calibrated value because it resulted in low values of both $R_{norm,error}$ and $A_{ratio,error}$ (< 3%) (Fig 5e-f, Fig 7b).

S9.4 Error propagation

To determine the uncertainty of R_{error}, propagation of errors was performed in MATLAB to generate the error bars in Fig 7b. The equation for propagation of error is:

$$\delta R_{error} = \sqrt{\left(\frac{\partial R_{error}}{\partial R_{sim}}\delta R_{sim}\right)^2 + \left(\frac{\partial R_{error}}{\partial R_{expt}}\delta R_{expt}\right)^2} \qquad \text{(S9.2)}$$

where δR_{error}, δR_{sim}, and δR_{expt} are the uncertainties of R_{error}, R_{sim}, and R_{expt} respectively. For equation S9.3, this evaluates to:

$$\delta R_{error} = \sqrt{\left(\frac{R_{sim} - R_{expt}}{R_{expt}\left|R_{sim} - R_{expt}\right|}\delta R_{sim}\right)^2 + \left(\frac{\left(-\left(R_{sim} - R_{expt}\right)^2 - R_{sim}R_{expt} + R_{expt}^2\right)}{R_{expt}^2\left|R_{sim} - R_{expt}\right|}\delta R_{expt}\right)^2} \qquad \text{(S9.3)}$$

Table S9.1. Experimental design for FFD experiment.

Raw Parameter Value			Coded Parameter Value			A_{ratio}		R_{norm}	
k_{mit}^{Adh}	k_{mit}^{Stiff}	P	k_{mit}^{Adh}	k_{mit}^{Stiff}	P	**Mean**	**Std**	**Mean**	**Std**
0	50	6.1	-1	-1	-1	1.5	0.2	0.57	0.20
0	50	14.5	-1	-1	0.08	3.1	0.5	0.53	0.26
0	50	21.7	-1	-1	1	6.8	1.7	0.25	0.54
0	275	6.1	-1	0	-1	1.2	0.1	0.79	0.10
0	275	14.5	-1	0	0.08	2.6	0.3	0.79	0.10
0	275	21.7	-1	0	1	5.4	0.9	0.79	1.93
0	500	6.1	-1	1	-1	1.1	0.1	0.85	0.09
0	500	14.5	-1	1	0.08	2.2	0.3	0.90	0.06
0	500	21.7	-1	1	1	4.7	0.9	0.89	0.11
15	50	6.1	0	-1	-1	1.7	0.2	0.14	0.21
15	50	14.5	0	-1	0.08	2.7	0.5	-0.12	0.58
15	50	21.7	0	-1	1	4.3	1.1	-1.36	1.61
15	275	6.1	0	0	-1	1.3	0.1	0.46	0.16
15	275	14.5	0	0	0.08	2.3	0.3	0.46	0.17
15	275	21.7	0	0	1	4.4	0.8	0.46	0.32
15	500	6.1	0	1	-1	1.1	0.1	0.61	0.14
15	500	14.5	0	1	0.08	2.2	0.3	0.65	0.11
15	500	21.7	0	1	1	3.9	0.6	0.68	0.21
30	50	6.1	1	-1	-1	1.5	0.2	-0.18	0.32
30	50	14.5	1	-1	0.08	2.3	0.4	-0.51	0.85
30	50	21.7	1	-1	1	3.6	1.7	-4.16	2.26
30	275	6.1	1	0	-1	1.2	0.1	0.25	0.21
30	275	14.5	1	0	0.08	2.1	0.3	0.25	0.29
30	275	21.7	1	0	1	3.7	0.7	0.03	0.75
30	500	6.1	1	1	-1	1.1	0.1	0.43	0.21
30	500	14.5	1	1	0.08	2.0	0.3	0.47	0.23
30	500	21.7	1	1	1	3.6	0.7	0.46	0.32

Table S9.2. Summary of regression model for Area ratio in FFD experiment.

	ANOVA					Coefficients			
Source	**Sum of Squares**	**df**	**Mean Square**	**F Value**	**p Value**	**Estimate**	**SEM**	**tStat**	**pValue**
Intercept						2.15	0.15	14.15	7.8E-11
A-k_{mit}^{Stiff}	3.13	1	3.13	35.35	1.6E-05	-0.4	0.07	-5.75	2.3E-05
B-k_{mit}^{Adh}	1.58	1	1.58	17.86	5.7E-04	-0.29	0.07	-4.19	6.1E-04
C-P	45.26	1	45.26	510.44	4.0E-14	1.6	0.07	22.8	3.5E-14
AB	0.58	1	0.58	6.52	0.02	0.22	0.09	2.55	0.02
AC	3.1	1	3.1	34.92	1.7E-05	-0.51	0.09	-5.91	1.7E-05
BC	0.09	1	0.09	1.06	0.32	-0.09	0.09	-1.03	0.32
A^2	0.07	1	0.07	0.82	0.38	0.11	0.12	0.91	0.38
B^2	1.0E-02	1	1.0E-02	0.11	0.74	0.04	0.12	0.34	0.74
C^2	2.34	1	2.34	26.41	8.2E-05	0.63	0.12	5.14	8.2E-05
Error	1.51	17	0.09	1	0.5				

R^2: 0.974, Adjusted R^2: 0.960, F-statistic vs. constant model: 70.4, p-value = 1.12e-11

Table S9.3. Summary of regression model for Roundness in FFD experiment.

	ANOVA					Coefficients			
Source	**Sum of Squares**	**df**	**Mean Square**	**F Value**	**p Value**	**Estimate**	**SEM**	**tStat**	**pValue**
Intercept						0.7	0.28	2.5	0.02
A-k_{mit}^{Stiff}	4.81	1	4.81	15.8	9.8E-04	-0.51	0.13	-3.91	1.1E-03
B-k_{mit}^{Adh}	6.47	1	6.47	21.24	2.5E-04	0.59	0.13	4.51	3.1E-04
C-P	1.83	1	1.83	6.01	0.03	-0.33	0.13	-2.51	0.02
AB	2.01	1	2.01	6.61	0.02	0.41	0.16	2.57	0.02
AC	1.2	1	1.2	3.93	0.06	-0.32	0.16	-1.98	0.06
BC	2.82	1	2.82	9.26	7.4E-03	0.48	0.16	3.04	7.4E-03
A^2	6.5E-03	1	6.5E-03	0.02	0.89	-0.03	0.23	-0.15	0.89
B^2	1.03	1	1.03	3.37	0.08	-0.41	0.23	-1.84	0.08
C^2	0.52	1	0.52	1.72	0.21	-0.3	0.23	-1.31	0.21
Error	5.18	17	0.3	1	0.5				

R^2: 0.800, Adjusted R^2: 0.694, F-statistic vs. constant model: 7.55, p-value = 2e-4

Table S9.4. Experimental design for CCD experiment.

Raw Parameter Value			Coded Parameter Value			A_{ratio}		R_{norm}	
k_{mit}^{Adh}	k_{mit}^{Stiff}	P	k_{mit}^{Adh}	k_{mit}^{Stiff}	P	**Mean**	**Std**	**Mean**	**Std**
1.6	450	21.0	-1	0	0.08	4.2	0.7	0.88	0.10
3.0	300	19.0	-0.76	-0.76	-0.74	4.0	0.5	0.81	0.13
3.0	300	23.0	-0.76	-0.76	0.89	5.3	0.8	0.79	0.16
3.0	600	19.0	-0.76	-0.76	-0.74	3.5	0.5	0.90	0.07
3.0	600	23.0	-0.76	-0.76	0.89	4.4	0.8	0.91	0.09
7.5	253	21.0	0	-1	0.08	4.3	0.7	0.70	0.20
7.5	450	18.4	0	0	-1.00	3.4	0.5	0.79	0.10
7.5	450	21.0	0	0	0.08	4.0	0.6	0.81	0.11
7.5	450	21.0	0	0	0.08	4.1	0.6	0.83	0.12
7.5	450	21.0	0	0	0.08	4.1	0.6	0.80	0.13
7.5	450	21.0	0	0	0.08	4.1	0.6	0.81	0.13
7.5	450	21.0	0	0	0.08	4.0	0.6	0.81	0.13
7.5	450	23.3	0	0	1	4.8	0.9	0.81	0.15
7.5	647	21.0	0	1	0.08	3.7	0.6	0.87	0.09
12.0	300	19.0	0.76	-0.76	-0.74	3.5	0.5	0.58	0.26
12.0	300	23.0	0.76	-0.76	0.89	4.6	0.7	0.65	0.28
12.0	600	19.0	0.76	-0.76	-0.74	3.3	0.5	0.79	0.12
12.0	600	23.0	0.76	-0.76	0.89	4.1	0.7	0.81	0.12
13.4	450	21.0	1	0	0.08	3.8	0.7	0.68	0.26

Table S9.5. Additional points for CCD experiment.

Raw Parameter Value			Coded Parameter Value			A_{ratio}		R_{norm}	
k_{mit}^{Adh}	k_{mit}^{Stiff}	P	k_{mit}^{Adh}	k_{mit}^{Stiff}	P	**Mean**	**Std**	Mean	Std
4.0	350	19.1	-0.59	-0.51	-0.70	4.0	0.8	0.60	0.17
4.0	450	19.1	-0.59	0.00	-0.70	3.9	0.8	0.58	0.11
7.2	322	21.0	-0.05	-0.65	0.08	4.3	0.7	0.65	0.20
7.4	330	21.0	-0.02	-0.61	0.08	4.4	0.7	0.67	0.16
7.7	340	21.0	0.03	-0.56	0.08	4.2	0.7	0.68	0.19
8.0	316	19.2	0.08	-0.68	-0.65	3.3	0.7	0.45	0.15
8.0	400	19.2	0.08	-0.25	-0.65	3.1	0.8	0.46	0.11
8.0	400	19.2	0.08	-0.25	-0.65	3.2	0.7	0.44	0.13
8.0	400	19.8	0.08	-0.25	-0.40	4.1	0.8	0.71	0.16
8.0	484	19.2	0.08	0.17	-0.65	3.2	0.8	0.38	0.10
12.0	350	19.1	0.76	-0.51	-0.70	3.8	0.6	0.62	0.30
12.0	450	19.1	0.76	0.00	-0.70	3.6	0.7	0.49	0.17

Table S9.6. Summary of regression model for Area ratio in CCD experiment.

	ANOVA					Coefficients			
Source	**Sum of Sq.**	**df**	**Mean Square**	**F Value**	**p Value**	**Estimate**	**SEM**	**tStat**	**pValue**
Intercept						3.97	0.07	53.29	6.7E-24
A-k_{mit}^{Stiff}	0.49	1	0.49	9.74	5.2E-03	-0.24	0.08	-3.1	5.4E-03
B-k_{mit}^{Adh}	0.94	1	0.94	18.74	3.0E-04	-0.34	0.08	-4.34	2.9E-04
C-P	4.62	1	4.62	91.63	4.1E-09	0.66	0.07	9.32	6.5E-09
AB	0.05	1	0.05	0.97	0.33	0.13	0.13	0.99	0.33
AC	7.5E-03	1	7.5E-03	0.15	0.7	-0.04	0.11	-0.39	0.7
BC	0.1	1	0.1	1.94	0.18	-0.17	0.12	-1.39	0.18
A^2	0.06	1	0.06	1.2	0.29	0.14	0.13	1.09	0.29
B^2	1.7E-03	1	1.7E-03	0.03	0.86	-0.03	0.14	-0.18	0.86
C^2	3.6E-04	1	3.6E-04	7.1E-03	0.93	0.01	0.13	0.08	0.93
Error	1.06	21	0.05	1	0.5				

R^2: 0.990, Adjusted R^2: 0.980, F-statistic vs. constant model: 98.8, p-value = 6.48e-8

Table S9.7. Summary of regression model for Roundness in CCD experiment.

	ANOVA					Coefficients			
Source	**Sum of Squares**	**df**	**Mean Square**	**F Value**	**p Value**	**Est.**	**SEM**	**tStat**	**pValue**
Intercept						0.8	4.9E-03	165	3.6E-34
A-k_{mit}^{Stiff}	0.09	1	0.09	410.4	2.9E-15	-0.1	5.1E-03	-19.0	1.0E-14
B-k_{mit}^{Adh}	0.07	1	0.07	344.4	1.7E-14	0.09	5.2E-03	18.32	2.2E-14
C-P	1.9E-03	1	1.9E-03	8.68	7.7E-03	0.01	4.6E-03	2.76	0.01
AB	4.1E-03	1	4.1E-03	19.16	2.6E-04	0.04	8.7E-03	4.38	2.6E-04
AC	1.7E-03	1	1.7E-03	7.99	0.01	0.02	7.4E-03	2.83	0.01
BC	8.3E-05	1	8.3E-05	0.38	0.54	-4.9E-3	7.8E-03	-0.62	0.54
A^2	1.6E-03	1	1.6E-03	7.6	0.01	-0.02	8.7E-03	-2.76	0.01
B^2	1.3E-03	1	1.3E-03	6	0.02	-0.02	9.0E-03	-2.45	0.02
C^2	4.2E-05	1	4.2E-05	0.19	0.67	-3.7E-3	8.4E-03	-0.44	0.67
Error	4.5E-03	21	2.2E-04	1	0.5				

R^2: 0.987, Adjusted R^2: 0.974, F-statistic vs. constant model: 75.6, p-value = 2.12e-7

References

1. Whitcomb PJ, Anderson MJ. RSM Simplified: Optimizing Processes Using Response Surface Methods for Design of Experiments. CRC Press; 2004.

2. DiStefano III J. Dynamic systems biology modeling and simulation [Internet]. Academic Press; 2015. Available: https://books.google.com/books?hl=en&lr=&id=nWoYAgAAQBAJ&oi=fnd&pg=PP1&dq=Dynamic+Systems+Biology+Modeling+and+Simulation+&ots=eCSrzd3r2V&sig=lcxCgeFWBE8NNk3rscT-8VTl9g0

3. Zartman J, Restrepo S, Basler K. A high-throughput template for optimizing Drosophila organ culture with response-surface methods. Development. 2013;140: 667–674. doi:10.1242/dev.088872

4. Narciso C, Wu Q, Brodskiy P, Garston G, Baker R, Fletcher A, et al. Patterning of wound-induced intercellular Ca 2+ flashes in a developing epithelium. Phys Biol. 2015;12: 056005. doi:10.1088/1478-3975/12/5/056005

5. Heller D, Hoppe A, Restrepo S, Gatti L, Tournier AL, Tapon N, et al. EpiTools: An Open-Source Image Analysis Toolkit for Quantifying Epithelial Growth Dynamics. Dev Cell. 2016;36: 103–116. doi:10.1016/j.devcel.2015.12.012
