# Supplementary material for: Multi-scale computational study of the mechanical regulation of cell mitotic rounding in epithelia
Source: PLoS Comput Biol. 2017 May 22;13(5):e1005533. doi: 10.1371/journal.pcbi.1005533 (PMC5460904; doi:10.1371/journal.pcbi.1005533)
Supplement: S10 Appendix — (PDF) [file pcbi.1005533.s010.pdf]

S10 Appendix: Stepwise regression for model reduction

The MATLAB function *stepwiselm* was used to perform stepwise regression to remove insignificant terms from model (Table S10.1-S10.4). The term with the largest p-value was iteratively removed from the model, and the model was refit. This was done until an F-test for change in the sum of squared error returned a p-value below α, which was set to be determined based on the impact on the adjusted R^2 of the model (Fig. S10.1). This was done to avoid over fitting the model.

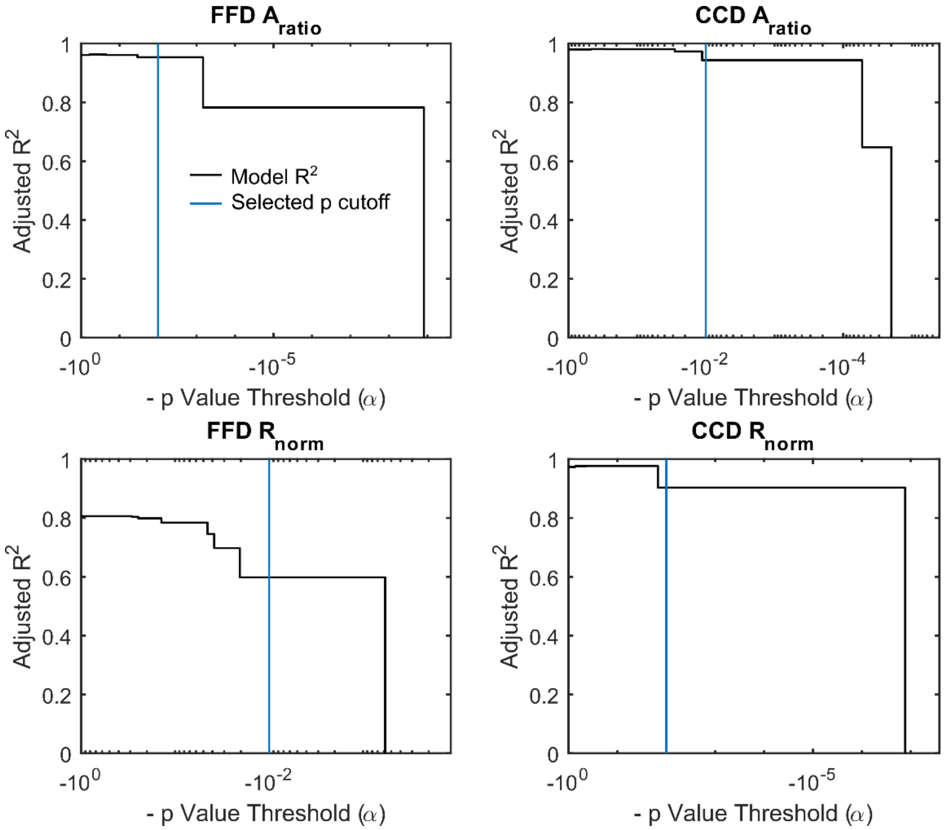

Fig S10.1. Selection of significance threshold for model reduction. A stepwise regression algorithm was used to reduce model complexity. A stepwise regression algorithm was used to reduce model complexity. The model term with the highest p-value was removed, and a t-test was used to determine whether the SEM of the fit was significantly reduced. If the p-value was greater than the threshold alpha, the term was removed and the model was refit. If not, then the process ended. Selection of alpha balances the cost of poor fit due to too few model terms, with the chance of overfitting caused by too many model terms. The adjusted R^2 of each of the four models is reported as a function of alpha. The alphas selected for this study are indicated by a blue line.

The reduced models for the CCD (Experiment 2) took the form:

$$\hat{A}_{ratio} = \beta_0 + \beta_A k_{mit}^{Adh} + \beta_B k_{mit}^{Stiff} + \beta_C \Delta P \quad (S10.1)$$

$$\hat{R}_{norm} = \beta_0 + \beta_A k_{mit}^{Adh} + \beta_B k_{mit}^{Stiff} + \beta_{AB} k_{mit}^{Adh} k_{mit}^{Stiff} \quad (S10.2)$$

Table S10.1. Summary of reduced regression model for A_{ratio} in FFD experiment.

	ANOVA					Coefficients			
Source	**Sum of Sq.**	**df**	**Mean Square**	**F Value**	**p Value**	**Estimate**	**SEM**	**tStat**	**pValue**
Intercept						1.26	0.42	2.98	7.1E-03
A-k_{mit}^{Stiff}	3.13	1	3.13	29.09	2.4E-05	0.03	0.01	2.67	0.01
B-k_{mit}^{Adh}	1.58	1	1.58	14.7	9.6E-04	-1.3E-03	3.4E-04	-3.83	9.6E-04
C-P	45.26	1	45.26	420.11	2.3E-15	-0.02	0.06	-0.28	0.78
Error	3.1	1	3.1	28.74	2.6E-05	-4.3E-03	8.1E-04	-5.36	2.6E-05

R^2: 0.961, Adjusted R^2: 0.951, F-statistic vs. constant model: 103, p-value = 4.9e-14

Table S10.2. Summary of reduced regression model for R_{norm} in FFD experiment.

	ANOVA					Coefficients			
Source	**Sum of Squares**	**df**	**Mean Square**	**F Value**	**p Value**	**Estimate**	**SEM**	**tStat**	**pValue**
Intercept						-0.02	0.33	-0.05	0.96
A-k_{mit}^{Stiff}	4.81	1	4.81	7.91	9.6E-03	-0.03	0.01	-2.81	9.6E-03
B-k_{mit}^{Adh}	6.47	1	6.47	10.64	3.3E-03	2.7E-03	8.2E-04	3.26	3.3E-03
Error	14.6	24	0.61	1	0.5				

R^2: 0.436, Adjusted R^2: 0.389, F-statistic vs. constant model: 9.28, p-value = 1.0e-3
The poor adjusted R^2 for the reduced model suggests that the full model is necessary (criterion; p<0.01).

Table S10.3. Summary of reduced regression model for A_{ratio} in CCD experiment.

	ANOVA					Coefficients			
Source	**Sum of Squares**	**df**	**Mean Square**	**F Value**	**p Value**	**Estimate**	**SEM**	**tStat**	**pValue**
Intercept						-0.69	0.58	-1.18	0.25
A-k_{mit}^{Stiff}	0.5	1	0.5	10.44	3.2E-03	-0.04	0.01	-3.23	3.2E-03
B-k_{mit}^{Adh}	0.9	1	0.9	19.06	1.7E-04	-1.7E-03	3.8E-04	-4.37	1.7E-04
C-P	4.83	1	4.83	101.82	1.2E-10	0.28	0.03	10.09	1.2E-10
Error	1.28	27	0.05	1	0.5				

R^2: 0.825, Adjusted R^2: 0.806, F-statistic vs. constant model: 42.5, p-value = 2.3e-10

Table S10.4. Summary of reduced regression model for R_{norm} in CCD experiment.

	ANOVA					Coefficients			
Source	**Sum of Squares**	**df**	**Mean Square**	**F Value**	**p Value**	**Estimate**	**SEM**	**tStat**	**pValue**
Intercept						0.82	0.04	20.89	3.4E-18
A-k_{mit}^{Stiff}	0.09	1	0.09	208.34	3.3E-14	-0.03	4.7E-03	-6.91	2.0E-07
B-k_{mit}^{Adh}	0.07	1	0.07	172.96	3.0E-13	2.1E-04	8.7E-05	2.45	0.02
AB	4.9E-03	1	4.9E-03	11.32	2.3E-03	3.5E-05	1.0E-05	3.36	2.3E-03
Error	0.01	27	4.3E-04	1	0.5				

R^2: 0.936, Adjusted R^2: 0.929, F-statistic vs. constant model: 132, p-value = 3.1e-16

The high adjusted R^2 value suggests that model reduction is reasonable for the parameter range tested for the CCD experiment.
