# Supplementary material for: Multi-scale computational study of the mechanical regulation of cell mitotic rounding in epithelia
Source: PLoS Comput Biol. 2017 May 22;13(5):e1005533. doi: 10.1371/journal.pcbi.1005533 (PMC5460904; doi:10.1371/journal.pcbi.1005533)
Supplement: S11 Appendix — (PDF) [file pcbi.1005533.s011.pdf]

S11 Appendix: Sensitivity analysis

Local sensitivity analysis was performed for parameter values at the center of the CCD to quantitatively predict the relative contributions of k_{mit}^{Adh}, k_{mit}^{Stiff}, and ΔP_{mit} to A_{ratio} and R_{norm} under experimental conditions. Sensitivity is calculated as follows [1]:

$$S_i^{y(\boldsymbol{x})} = \frac{\partial y(\boldsymbol{x})}{\partial x_i} \frac{x_i}{y(\boldsymbol{x})} \quad \text{(S11.1)}$$

where $S_i^{y(\boldsymbol{x})}$ is the sensitivity, which is the relative change of the response variable y to changes in parameter x_i when all other parameters ($\boldsymbol{x}_{i \neq i}$) are held constant.

In this study, all response variables were adequately modeled by a quadratic model, which for three parameters takes the form:

$$\hat{y} = \beta_0 + \beta_1 x_1 + \beta_2 x_2 + \beta_3 x_3 + \beta_{12} x_1 x_2 + \beta_{13} x_1 x_3 + \beta_{23} x_2 x_3 + \beta_{11} x_1^2 + \beta_{22} x_2^2 + \beta_{33} x_3^2 \quad \text{(S11.2)}$$

where $\hat{y}$ is the predicted value of y, and β's are fitted model coefficients.
For example, the sensitivity of the response $\hat{y}$ to the first parameter x_1 evaluates to:

$$S_1^{\hat{y}(\boldsymbol{x})} = (\beta_1 + \beta_{12} x_2 + \beta_{13} x_3 + 2\beta_{11} x_1) \frac{x_1}{\hat{y}(\boldsymbol{x})} \quad \text{(S11.3)}$$

The uncertainty of the sensitivity can be determined from the uncertainty of the model coefficients using the equation for propagation of uncertainty. *In silico* and specified model parameters do not have associated uncertainties. A sample uncertainty calculation follows:

$$\delta S_1^{\hat{y}(\boldsymbol{x})} = \sqrt{\left(\frac{\partial S_1^{\hat{y}(\boldsymbol{x})}}{\partial \beta_1} \delta\beta_1\right)^2 + \left(\frac{\partial S_1^{\hat{y}_1(\boldsymbol{x})}}{\partial \beta_{12}} \delta\beta_{12}\right)^2 + \left(\frac{\partial S_1^{\hat{y}_1(\boldsymbol{x})}}{\partial \beta_{13}} \delta\beta_{13}\right)^2 + \left(\frac{\partial S_1^{\hat{y}_1(\boldsymbol{x})}}{\partial \beta_{11}} \delta\beta_{11}\right)^2} \quad \text{(S11.4)}$$

$$\delta S_1^{\hat{y}(\boldsymbol{x})} = \frac{x_1}{\hat{y}(\boldsymbol{x})} \sqrt{\delta\beta_1^2 + (x_2 \delta\beta_{12})^2 + (x_3 \delta\beta_{13})^2 + (2x_1 \delta\beta_{11})^2} \quad \text{(S11.5)}$$

where $\delta S_1^{\hat{y}_1(\boldsymbol{x})}$, $\delta\beta_1$, $\delta\beta_{12}$, $\delta\beta_{13}$, and $\delta\beta_{11}$, represent the uncertainty of $S_1^{\hat{y}_1(\boldsymbol{x})}$, β_1, β_{12}, β_{13}, and β_{11} respectively.

References

1. DiStefano III J. Dynamic systems biology modeling and simulation [Internet]. Academic Press; 2015. Available: https://books.google.com/books?hl=en&lr=&id=nWoYAgAAQBAJ&oi=fnd&pg=PP1&dq=Dynamic+Systems+Biology+Modeling+and+Simulation+&ots=eCSrzd3r2V&sig=lcxCgeFWBE8NNk3rscT-8VTl9g0
