# Supplementary material for: Multi-scale computational study of the mechanical regulation of cell mitotic rounding in epithelia
Source: PLoS Comput Biol. 2017 May 22;13(5):e1005533. doi: 10.1371/journal.pcbi.1005533 (PMC5460904; doi:10.1371/journal.pcbi.1005533)
Supplement: S12 Appendix — (PDF) [file pcbi.1005533.s012.pdf]

S12 Appendix: Removal of an outlier

Although the Epi-Scale model is capable of modelling any cell shape, this study focuses on mitotic cells ranging from polygonal to circular shapes. The regression models used to interpret the results are able to robustly fit to these types of data. However, other cell shapes such as star-shaped cells were observed for high values of ΔP, low values of k_{mit}^{Stiff}, and high values of k_{mit}^{Adh}. One outlier over six median absolute variations from the mean was detected and removed (Fig S12.1).

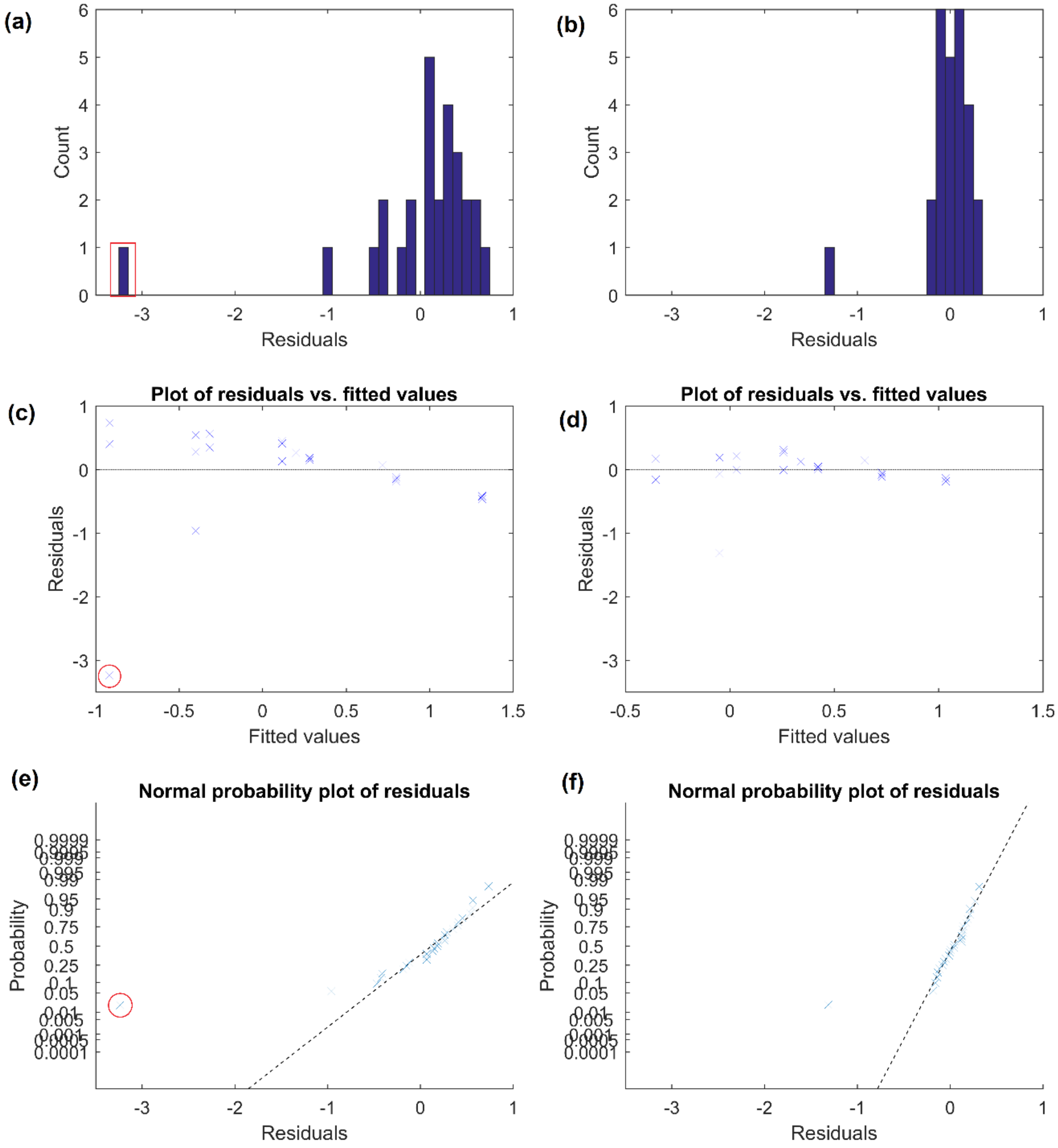

Fig S12.1. Outlier detection. One outlier over six median absolute variations from the mean was detected and removed. a) Histogram of residuals. b) Histogram of residuals with outlier removed. c) Plot of residuals vs. fitted values. d) Plot of residuals vs. fitted values with outlier removed. e) Normal probability plot of raw residuals. f) Normal probability plot of raw residuals with outlier removed.
